# Determinants of financial resilience: insights from an emerging economy

**DOI:** 10.1007/s40847-023-00239-y

**Published:** 2023-03-03

**Authors:** Fazelina Sahul Hamid, Yiing Jia Loke, Phaik Nie Chin

**Affiliations:** 1 Bristol Business School, University of the West of England, BS16 1QY Bristol, United Kingdom; 2 School of Social Science, Universiti Sains Malaysia, 11800 George Town, Penang Malaysia; 3 Graduate Business School, Universiti Sains Malaysia, 11800 George Town, Penang Malaysia

**Keywords:** Financial resilience, Financial inclusion, Financial literacy, Malaysia

## Abstract

The Organisation for Economic Co-operation and Development Financial Literacy Survey of 2018 response is used to study the impact of financial knowledge, financial inclusion, and socio-demographic characteristics on financial resilience. The measurement of financial resilience considers elements related to keeping control of money, taking care of expenditures, having a financial cushion, handling financial shortfall or stress, and having financial planning. Using a sample of 3395 individuals across Malaysia, we find that greater financial knowledge is associated with the probability of being financially resilient. Greater financial inclusion in terms of having more bank accounts and holding more financial products is linked to the probability of being financially resilient. We also find that financial resilience varies across certain socio-demographic characteristics. Implications of the findings are discussed.

## Introduction

Research related to the financial situation of households has gained momentum since the worsening of economic conditions in the recent years. Increasingly, more individuals are negatively affected during these challenging times. The recent Organisation for Economic Co-operation and Development (OECD) survey on 125,787 adults in twenty-six countries shows a worrying trend (OECD 2020a, b). Almost a third of the respondents only have savings that can last them for a week and almost half of the respondents acknowledge that they are concerned about ability in meeting their everyday living expenses. This shows that large number of individuals within many economies have limited ability in facing financial challenges. Lack of financial resilience do not just affect the households, but it also has a bearing on the economy at large. Many regulators and policy makers across the globe are concerned about it as less resilient individuals will have a greater tendency to rely on state for handouts. Moreover, lower financial resilience also may affect the overall stability of the financial system.

Having financial resilience is one of the major contributor to financial wellbeing (Russell et al. 2020). Financial resilience relates to individuals’ ability in coping with financial shock or recovering from financial difficulties (Mcknight and Rucci 2020). Individuals often face challenges in dealing with unexpected shocks such as illness, death of a family member, job loss or natural disaster. Individuals who have planned their finances well would rely on their savings during the difficult times. Alternatively, they may borrow money from financial institutions, family, or friends. Some may also rely on insurance payouts. Individuals who are not able to cope with financial challenges are classified as financially vulnerable (Lusardi et al. 2021).

This paper aims to investigate financial resilience in the context of an emerging economy. In doing so, it explores the multidimensional nature of the construct. Malaysia is chosen as it is one of the countries that is categorised as an upper middle-income nation. Absolute poverty eradication is not the major concern of the policy makers in the country as it has moved higher in the economic ladder. However, relative poverty remains a major issue that requires attention. The households in Malaysia face various types of financial challenges. The household debt to Gross National Product (GDP) of 93.4 percent in 2020 is the highest in the region. A higher debt level raises individuals’ vulnerability to adverse shock (Jappelli et al. 2013). Increasingly more Malaysians, especially those living in the urban areas, are struggling with rising cost of living. World Bank (2019) reports that there is a high degree of unaffordability among the low-income and middle-income households. Additionally, the report also states that household savings in Malaysia is low compared to OECD countries. These suggest that studying financial resilience from the context of an emerging economy like Malaysia is very relevant and timely as it can provide a different perspective about the topic.

In studying the state of financial resilience in Malaysia, this paper takes into consideration of the five components of financial resilience proposed by OECD (2020a; b)^1^ which includes elements related keeping control of money, taking care of expenditures, having a financial cushion, handling financial shortfall or stress and having a financial planning. These components enable us to identify the level of resilience among individuals by considering various aspects of personal financial management that are important for the financial wellbeing of individuals. We draw conclusion using the Organisation for Economic Co-operation and Development (International Network on Financial Education) Financial Literacy Survey. The 2018 OECD (INFE) Financial Literacy survey response is used to analyse financial resilience among Malaysians. Implications for research and policy are then presented.

This paper is organised as follows. “Literature review” section includes the literature review. “Data and methodology” section describes the methodology and the data used in this study. “Regression results” section presents the results while “Discussion and conclusion” section presents the discussion and conclusion of the study. The final section provides the implications of the findings.

## Literature review

Financial resilience is one of the aspects of resilience that has been investigated in the literature. The earlier study by Lusardi et al. (2011) has defined financial resilience as the individuals’ ability to raise emergency funds from various sources when needed. The theoretical background of precautionary motive can be derived from the Life-Cycle Hypothesis model of consumption and savings. This theory postulates that individuals save to smooth consumption over their lifetime. Modigliani and Brumberg (1954) postulates that savings enable individuals to face emergencies that may either arise due to a temporary reduction in income or unexpected increase in expenses. In contrast, Muir et al. (2016) and Salignac et al. (2019) define financial resilience as the ability of individuals in relying on their internal and external resources during adverse shock. The internal resource refers to individuals’ ability in managing their finances by saving and taking care or their expenses, while external resource refers to the reliance on family, friends, or other form of social support during a financial shock.

Salignac et al. (2019) argue that focusing only on the individual’s ability in managing financial shock is not sufficient as it takes into consideration of the other aspects such as context, structures and supports. They develop a framework that helps us understand financial resilience from the viewpoint of financial inclusion. Their framework incorporates four interconnected factors related to economic resources; financial products and services; financial knowledge and behaviour and social capital. Similarly, Goyal et al. (2021) incorporated individuals reliance on both internal and external resources in studying financial resilience among Indians during the COVID-19 pandemic.

Meanwhile, OECD (2020a; b) conceptualisation of financial resilience incorporates elements related to keeping control of money, taking care of expenditures, having a financial cushion, handling financial shortfall or stress, having a financial planning and being aware of fraud. This conceptualisation of financial resilience corresponds mainly to the individual’s ability in assessing and using internal resources when facing financial challenges. These elements mostly relate to the economic resource factor in the study by Salignac et al. (2019).

Kass-Hanna et al. (2021) and Lusardi et al. (2021) studied the role of financial literacy in influencing financial resilience. Kass-Hanna et al. (2021) find that higher financial literacy is linked to more saving, more borrowing, better risk management behaviours related to having life and health insurance and better emergency preparedness. Lusardi et al. (2021) observe that higher financial literacy is linked to better emergency preparedness, less debt constrains, better planning for the future and greater tendency to save and plan for retirement.

The role of financial inclusion in influencing financial resilience has also been studied in the literature. Even though the definition of financial inclusion varies in the literature, most studies have defined it in terms of the comprehensive financial product holdings (Lyons and Kass-Hanna 2021). However, initial studies focused more on basic account access (Cihak et al. 2016). Salignac et al. (2021) asserts that financial inclusion enables access to suitable and reasonable financial product and services. This facilitates investment in various aspects including business, education and health. Financially excluded individuals have a higher tendency in engaging in informal financing that is often more costly (Lamb 2016). Kass-Hanna et al. (2021) state that having financial products related to savings, loans and insurance allows individuals to make more strategic financial decisions that can mitigate risk and allow individuals to be more prepared to face financial shock. Based on the development economic perspective, Salignac et al. (2021) postulate that financial inclusion can serve as a catalyst that stimulates economic development since countries that are low in financial inclusion often have higher poverty rate, greater income inequality and lower economic growth. Belayeth Hussain et al. (2019) observed greater resilience among bank account holders compared to non-account holders, whereas Lyons and Kass-Hanna (2021) finds that greater financial inclusion is linked to lower financial vulnerability.

Additionally, studies have also scrutinised the role of socio-demographic characteristics in influencing financial resilience. Belayeth Hussain et al. (2019), Kass-Hanna et al. (2021), Lusardi et al. (2011), and Salignac et al. (2021) find that financial resilience varies across gender, income level, education level, employment status, regions, urbanisation, ethnicity, and number of dependent in the household. There are limited studies that have looked at financial resilience among Malaysians. Most of the studies on financial management among Malaysians have focused on issues related to financial preparedness (Loke 2016), financial wellbeing (Mahdzan et al. 2019), financial planning (Boon et al. 2011), financial literacy (Yew et al. 2017), and debt repayment behaviour (Hamid and Loke 2021). This study aims to fill the gap in the literature by identifying the determinants of financial resilience among Malaysians.

**Fig. 1:**
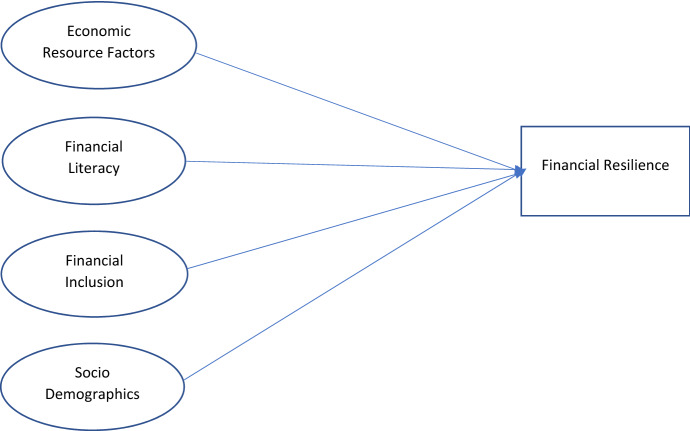
Conceptual framework

Considering the above discussions, Fig. 1 shows the conceptual framework of this study. Firstly, we would like to confirm whether economic resource factors influence financial resilience. Secondly, we would like to identify effect of financial literacy on financial resilience. Thirdly, we would like to study how financial inclusion influences financial resilience. Lastly, we would like to consider the effect of social demographic variables on financial resilience.

## Data and methodology

### Data

The data used in this study are provided by the Bank Negara Malaysia, the central bank of Malaysia. It is sourced from the OECD (INFE) Financial Literacy survey that was collected in 2018. The survey was conducted nationwide, covering the East and West of Malaysia. A total of 3,394 individuals participated in the study. The survey included questions on financial literacy which incorporates financial knowledge, behaviour, and attitudes. In addition, the survey also included questions related to money management, short- and long-term financial planning and financial product choice. Questions related to the socio-demographic characteristics of respondents were also included in the survey.

### Variables

#### Dependent variable

Financial resilience is a concept that is dynamic as it incorporates the individuals’ ability to recover from an adverse financial shock. In practise, longitudinal data are required to capture the individuals’ financial condition when the shock happens and observe their ability in dealing with it in the subsequent years. However, it is time consuming and costly to carry out such analysis. As such, cross-sectional analysis is mostly used in studying individuals’ financial resilience. In line with OECD (2020a; b), five components of financial resilience are considered. The survey questions used to measure each component of the financial resilience are included in Table 1. The first component (Resil 1) is related to keeping control of money which includes elements associated with planning financial matters and keeping a budget. This component is measured using three questions with a total score ranging from 3 to 11. The second component (Resil 2) is related to taking care of expenditure assesses elements linked to managing expenses and fulfilling financial obligations on time to ensure that no late charges or penalty are incurred. This component is estimated using two questions with a total score ranging from 2 to 10. The third component (Resil 3) is related to having financial cushion, taking into consideration elements connected to the ability in withstanding financial shock. This component is analysed using two questions with a total score ranging from 2 to 7. The fourth component (Resil 4) is related to handling financial stress accounts for elements that considers experiencing financial shortfall and stress related to financial matters. This component is estimated using three questions with a total score ranging from 3 to 13. The last component (Resil 5) is related to having a financial planning includes elements linked to actively saving and having financial goals in the future. This component is analysed using three questions with a total score ranging from 3 to 12.

**Table 1:** Financial resilience components

Construct	Questions	Answer and Score
Resil 1—Keeping control of money	I keep a close personal watch on my financial affairs Before receiving money, do you plan on how the money will be used? Does your household have a budget?	Answer: Completely disagree to completely agree Score: 1 to 5 Answer: No, sometimes, always Score: 1 to 3 Answer: No, sometimes, always Score: 1 to 3 Total score: 3 to 11
Resil 2—Taking care of expenditures	Before I buy something I carefully consider whether I can afford it I pay my bills on time	Answer: Completely disagree to completely agree Score: 1 to 5 Answer: Completely disagree to completely agree Score: 1 to 5 Total score: 2 to 10
Resil 3—Having a financial cushion	How easy is it for you to get emergency cash of RM1000? If tomorrow you had to meet a major unexpected expense of at least a month salary/income, could you cover it in full and without borrowing money that you would have to repay?	Answer: Extremely difficult to extremely easy Score: 1 to 5 Answer: No or Yes Score: 1 to 2 Total score: 2 to 7
Resil 4—Handling financial shortfall or stress	How straining is the debt that you owe? I tend to worry about paying my normal living expenses Do you run short of money for food or other necessary items?	Answer: Extremely straining to not straining at all Score: 1 to 5 Answer: Completely agree to completely disagree Score: 1 to 5 Answer: Regularly, sometimes or no Score: 1 to 3 Total score: 3 to 13
Resil 5—Having a financial planning	I try to save some money regularly, even if it is only a little Do you have any short or medium-term financial goals? I set long-term financial goals and strive to achieve them	Answer: Completely disagree to completely agree Score: 1 to 5 Answer: No or Yes Score: 1 to 2 Answer: Completely disagree to completely agree Score: 1 to 5 Total score: 3 to 12

Based on the 13 questions used, the raw value of Total Resilience will range from 13 to 53. However, the range of scores is not uniform across each component. As such, respondents score for each component is estimated by dividing the score for each component with the total value of each component, and multiplying the sum obtained by 53 (the maximum score of Total Resilience). The weighted value for Total Resilience is estimated by adding the weighted scores from each of the five components. In doing so, we have assumed that each component contributes equally to the financial resilience.^,^^2^,^3^ The weighted values of component of financial resilience ranges from 2.12 to 10.6. Based on these values, each component’s score is classified into three categories: low, medium, and high. This is done by dividing the range into three groups. The score between 2.12 and 4.95 is classified as low (0), the score between 4.96 and 7.79 is classified as medium (1), and the score between 7.8 and 10.6 is classified as high (2). Meanwhile, the weighted value for Total Resilience ranges from 13.22 to 53. Based on these values, the Total Resilience score is divided into three categories: low, medium, and high. The Total Resilience score between 13.22 and 26.48 is classified as low (0), the score between 26.49 and 39.75 is classified as medium (1), and the score between 39.76 and 53 is classified as high (2).

Table 2 presents the distribution of the respondents into the three categories for Total Resilience and five components. 25.37 percentage of the respondents falls into the low category of Total Resilience, which is considered as financially vulnerable. Almost half of the respondents (51.09 percent) fall into the medium category for Total Resilience, displaying moderate financial resilience. On the other hand, 23.54 percent of the respondents belong to the high category group of Total Resilience, which is considered as financially resilient. As far as the components of financial resilience are concerned, only 18.77 percent of the respondents fall into the low category group for Resil 1. The remaining 42.1 and 39.13 percent consist of those who belong to the medium and high category group for Resil 1, respectively. This demonstrates that those who are good at keeping control of money are almost double compared to those who are not. Additionally, it is observed that almost half of the respondents (50.15 percent) are good at taking care of the expenditure (Resil 2). Only 15.67 percentage of the respondents are weak at managing their expenditure, while the remaining 34.18 percent are moderate at it. When it comes to having financial cushion (Resil 3), it is noticed that 44.81 percent of the respondents are weak at it, 50.85 percent are moderate at it, and only 4.33 percent are good at it. As far as handling financial stress (Resil 4) is concerned, majority of the respondents (52.3 percent) are moderate at it. The remaining 36.8 percent of the respondents are good at handling financial stress, while only 10.9 percent of the respondents are weak at it. Finally, it is observed that majority of the respondents (51.97 percent) are good at financial planning (Resil 5). Those who are weak in financial planning account for 22.98 of the respondents, while the balance 25.04 percent are moderate at it.

**Table 2:** Distributions of dependent variables

	Count of respondents	Percentage of total sample
Total resilience		
Low	861	25.37
Medium	1734	51.09
High	799	23.54
Resil 1		
Low	637	18.77
Medium	1429	42.1
High	1328	39.13
Resil 2		
Low	532	15.67
Medium	1160	34.18
High	1702	50.15
Resil 3		
Low	1521	44.81
Medium	1726	50.85
High	147	4.33
Resil 4		
Low	370	10.9
Medium	1775	52.3
High	1249	36.8
Resil 5		
Low	780	22.98
Medium	850	25.04
High	1764	51.97

Resil 1 relates to keeping control of money; Resil 2 relates to taking care of expenditures; Resil 3 relates to having a financial cushion; Resil 4 relates to experiencing financial shortfall or stress and Resil 5 relates to having a financial planning

Additional analysis is done by using an alternative measure of financial resilience. Access to savings has been used by Mcknight and Rucci (2020) to study resilience. Even though it is just one of the elements of financial resilience proposed by Salignac et al. (2019), Mcknight and Rucci (2020) postulates that adequacy of saving is in itself is a good indicator to assess financial resilience since sufficient savings allow individuals to survive during challenging times without large borrowing. In assessing adequacy of saving, respondents were asked how much of their current earnings do they save. The response is classified into five categories. The categories range from less than 5 percent, 5 to 10 percent, 10 to 20 percent, 20 to 35 percent, 35 to 50 percent and above 50 percent. Individuals with higher savings are considered more resilient.

#### Explanatory variables

The explanatory variables used in the analyses are financial knowledge, financial inclusion, and socio-demographic variables. Financial knowledge is measured using seven questions related to time value of money, interest paid on a loan, simple interest calculation, compound interest calculation, risk and return, inflation, and risk diversification. For each question, respondents are allowed to choose any of the given answer or choose “I don’t know”. Each correct answer will be given a value of 1. Hence, the total value for financial knowledge ranges from 0 to 7. A value between 0 and 2 is considered as low financial knowledge, a value between 3 and 4 is considered moderate financial knowledge, and a value between 5 and 7 is considered high financial knowledge.

Financial inclusion is measured from two aspects: using bank products and services and level of financial product holding. Using bank products and services is classified into not using any banking products and services, using banking products and services from a single bank, and using banking products and services from several banks. Using products and services from several banks is used as the reference group. Level of financial product holdings consider respondents holding of deposit account, loan account, credit or debit card, insurance or takaful products, investment products and retirement products. Low product holding is classified as those holding two products and less, moderate product holding is classified as those holding three or four products and high product holding is classified as those holding five to six products.

Several socio-demographic characteristics are included as the control variables in the analyses. Gender is included in the analyses with value 1 assigned to male and 0 to female. Age is classified into six categories: 24 years and less; 25 to 29; 30 to 39; 40 to 49; 50 to 59; 60 years and above. Age category of 24 years and less is used as the reference group. Respondents’ education level is categorised into four, no formal education or primary education, secondary education, vocational education beyond secondary school and university education. Having university education is used as the reference group. Income is divided into four categories which are RM1500 and less; RM1501 to RM5000; RM5001 to RM10,000 and above RM10,000. Earning income of RM1500 and less is used as the reference group. Ethnicities considered are Malays, Chinese, Indian and others, with Malays used as the reference group. Marital status in divided into single, married and divorced or widowed. Being married is used as the reference group. Dependents is classified into three categories based on whether the income is used to support oneself, immediate family, or extended family, with supporting oneself used as the reference group.

According to World Bank (2021), there are economic disparity among the regions in Malaysia. In line with that, region of residence is included in the analysis. Peninsula Malaysia is represented by four regions which includes the northern, central, eastern, and southern regions. East Malaysia is considered the fifth region. The central region of Peninsula Malaysia is used as the reference group. Additionally, location of residence in terms of whether the respondent resides in a city centre, urban or rural area are also controlled for with living in city centre used as the reference group.

## Econometric specification

The ordered logistic regression was used to identify the determinants five components of resilience and the Total Resilience. This regression model is appropriate when the dependent variable consist of choices between more than two options and these choices display a natural order of options (Cameron and Trivedi 2010). The following ordered logit model is used:

1 $$y* = \beta^{\prime } x + \varepsilon$$

Based on the above equation, y* is a latent variable that is not directly observed. It is a continuous measure of financial resilience level that is coded as 0, 1 and 2, whereas *x* is the vector of explanatory variables that takes into account of financial knowledge, financial inclusion and socio-demographics factors. The *β*′s are the related coefficient, and *ε* is the error term.

The discrete financial resilience level variable that is observed, y, is derived as following from the model:

2 $$y = 0\;\left( {{\text{low}}} \right),\;{\text{if}}\;y* < \mu_{1}$$

3 $$y = 1\left( {{\text{moderate}}} \right),\;{\text{if}}\;\mu_{1} < y* < \mu_{2}$$

4 $$y = 2\left( {{\text{high}}} \right),\;{\text{if}}\;y* > \mu_{2}$$

where $\mu_{1}$ and $\mu_{2}$ are threshold variables in the logit model. The threshold variables are unknown and determine the maximum likelihood estimation procedure for the ordered logit. Using the cumulative normal function, the probability for each level of financial resilience is:

5 $$\Pr \left( {y = 0} \right) = \varphi \left( { - \beta^{\prime } x} \right)$$

6 $$\Pr \left( {y = 1} \right) = \varphi \left( { - \beta^{\prime } x} \right) - \varphi \left( { - \beta^{\prime } x} \right)$$

7 $$\Pr \left( {y = 2} \right) = 1 - \varphi \left( { - \beta^{\prime } x} \right)$$

The explanatory variables used in this study are all categorical variables. All the ordered logit estimation was done using robust standard errors.

## Descriptive statistics

Table 3 presents the distribution of Total Resilience based on financial knowledge, financial inclusion, and socio-demographics characteristics of the respondents. With regard to financial knowledge, 37.89 percent of the respondents have higher financial knowledge, 36.56 percent have moderate financial knowledge and 25.55 percent have lower financial knowledge. A lower percentage of those with low Total Resilience have high financial knowledge. Meanwhile, a higher percentage of those with high Total Resilience have high financial knowledge. When it comes to financial inclusion, we find that more than half of the respondents only uses products and services of a single bank. 36.92 percent of respondents uses product and services of several banks, while 10.58 percent of the respondents do not have any bank account. It is observed that those a higher percentage of those with low Total Resilience have a single bank account, while a higher percentage of those with high Total Resilience have several bank accounts. Almost half of the respondents only have a low level of financial products holding. The remaining 30.47 percent of the respondents have a moderate level of financial products holding and 19.68 percent have a high level of financial products holding. A lower percentage of those with low Total Resilience have a high level of products holding. Whereas a higher percentage of those with high Total Resilience have a moderate level of products holdings.

**Table 3:** Financial knowledge, financial inclusion and socio-demographic variable by total resilience

	Total resilience (%)			Total percentage
	Low	Medium	High	
Financial knowledge				
Low	9.99	13.02	2.53	25.55
Moderate	9.34	18.89	8.34	36.56
High	6.04	19.18	12.67	37.89
Financial inclusion				
Bank product and service usage				
No bank account	5.66	4.3	0.62	10.58
Single bank account	14.02	29.35	9.13	52.5
Several bank account	5.69	17.44	13.79	36.92
Financial products holding				
Less products holding	15.94	26.34	7.57	49.85
Moderate products holding	6.39	15.88	8.19	30.47
High products holding	3.03	8.87	7.78	19.68
Socio-demographics				
Gender				
Male	12.61	26.61	11.58	50.8
Female	12.76	24.48	11.96	49.2
Age				
Age 24 and less	11.58	15.79	3.39	30.76
Age 25 to 29	2.18	5.86	3.09	11.14
Age 30 to 39	3.98	11.05	6.45	21.48
Age 40 to 49	3.01	9.22	5.1	17.32
Age 50 to 59	3.48	7.1	4.21	14.79
Age 60 and above	1.15	2.06	1.3	4.51
Ethnicity				
Malay	15.29	29.2	12.96	57.45
Chinese	6.13	12.64	7.72	26.49
Indian	2.15	4.12	1.53	7.81
Others	1.8	5.13	1.33	8.25
Education				
Less than secondary	1.65	3.19	1	5.84
Secondary	19.79	37.99	13.24	71.03
Vocational	1.12	3.07	1.83	6.02
University	2.8	6.84	7.46	17.11
Marital status				
Single	13.97	20.81	6.75	41.53
Married	10.67	29.47	16.27	56.41
Divorced or widowed	0.74	0.83	0.5	2.06
Income				
Very low income	2.22	4.05	0.41	6.69
Low income	13.5	28.32	9.68	51.49
Middle income	6.6	13.14	8.17	27.91
High income	2.96	5.56	5.39	13.91
Location				
City centre	6.39	12.4	6.78	25.57
Urban	11.67	24.31	10.58	46.55
Rural	7.31	14.38	6.19	27.87
Region				
North Peninsula Malaysia	7.48	11.58	5.13	24.19
Centre Peninsula Malaysia	6.54	12.49	6.19	25.22
South Peninsula Malaysia	4.07	10.52	3.51	18.09
East Peninsula Malaysia	3.15	5.63	4.07	12.85
East Malaysia	4.12	10.87	4.66	19.65
Dependent				
Oneself	11.21	23.21	8.26	42.68
Immediate family	11.09	25.44	14.1	50.63
Extended family	2.56	2.71	1.42	6.69

The respondents are divided almost equally in terms of gender, with a higher percentage of both genders having a moderate Total resilience. The largest group of respondents in terms of age category are those aged 24 and less (30.76%) while the smallest group are those aged 60 and above (4.51%). A lower percentage of respondents in all age categories have high Total Resilience. The majority of the respondents are Malays (57.45%). This is followed by Chinese (26.49%), others (8.25%) and Indians (7.81%). A higher percentage of the respondents in all ethnic categories have moderate Total Resilience. Majority of the respondents only have secondary school education (71.03%). Respondents with university education accounts for 17.11 percent of the total respondents, vocational education accounts for 6.02 percent while those primary or no education accounts for 5.84 percent. A higher percentage of those with university degree have a high Total Resilience.

More than half of the respondents are married (56.41%), while 41.53 percent are single. A higher percentage of married and single individuals have moderate Total Resilience. Bulk of the respondents belongs to the low-income category (51.49%). This is followed by middle-income (27.91%), high-income (13.91%) and very low-income (6.69%). A lower percentage of individuals with very low or low income have high Total Resilience. Almost half of the respondents use their income to support themselves and their immediate family (50.63%), while 42.68 percent of the respondents only support themselves. Those who support themselves and their extended family accounts for 6.69 percent of the respondents. A higher percentage of those who support themselves and their immediate family have higher Total Resilience.

Additionally, that the data shows that 46.55 percent of respondents live in urban areas, 27.87 percent lives in rural areas and 25.57 percent lives in city centre. A lower percentage of those who live in rural area have a higher Total Resilience. As for the geographical location, 25.22 percent of the respondents are from the central region of Peninsula Malaysia, 24.19 percent are for the northern region, 12.85 percent are from the eastern region, and 18.09 percent are form the southern region. Those from East Malaysia accounts for 19.65 percent of the total respondents. A lower percentage of those living in the eastern region of Peninsula Malaysia and East Malaysia have a low Total resilience.

## Regression results

The ordered logit regression is used to analyse the relationship between the dependent variable (Total Resilience and five components of resilience) and independent variables (financial knowledge, financial inclusion, and socio-demographic characteristics). Table 4 shows the regression results for Total Resilience and all components of resilience. As can be seen, the coefficients of high and moderate financial knowledge are positive and significant. This indicates that, in comparison with having low financial knowledge, having moderate and higher financial knowledge increases the probability of being financially resilient and reduces the probability of being financially vulnerable. Similar results are observed for all components of financial resilience.

**Table 4:** Ordered logit regressions on the determinants of financial resilience

Variables	(1)	(2)	(3)	(4)	(5)	(6)
	Total resilience	Resil 1	Resil 2	Resil 3	Resil 4	Resil 5
	Coeff	Coeff	Coeff	Coeff	Coeff	Coeff
Fin knowledge moderate	0.583***	0.596***	0.255***	0.244**	0.017	0.664***
	(0.094)	(0.089)	(0.091)	(0.100)	(0.096)	(0.097)
Fin knowledge high	1.086***	0.428***	0.368***	0.188*	0.325***	0.746***
	(0.100)	(0.094)	(0.098)	(0.109)	(0.100)	(0.100)
Single bank account	− 0.508***	− 0.331***	− 0.010	− 0.213**	0.030	− 0.206**
	(0.080)	(0.078)	(0.078)	(0.083)	(0.076)	(0.080)
No bank account	− 1.272***	− 1.223***	− 0.694***	− 1.034***	− 0.017	− 0.895***
	(0.144)	(0.145)	(0.144)	(0.170)	(0.166)	(0.137)
Moderate product holdings	0.081	0.070	0.152*	− 0.018	0.136	− 0.032
	(0.086)	(0.081)	(0.083)	(0.089)	(0.086)	(0.085)
High product holdings	0.292***	0.263**	0.066	0.209*	− 0.119	− 0.001
	(0.108)	(0.107)	(0.107)	(0.115)	(0.099)	(0.111)
Gender	− 0.123*	− 0.314***	− 0.038	− 0.062	− 0.127*	− 0.061
	(0.069)	(0.068)	(0.069)	(0.075)	(0.069)	(0.069)
Age 25 to 29	0.390***	0.285**	0.490***	0.557***	− 0.179	0.147
	(0.132)	(0.131)	(0.127)	(0.140)	(0.130)	(0.134)
Age 30 to 39	0.410***	0.299**	0.327**	0.842***	− 0.126	0.140
	(0.137)	(0.136)	(0.131)	(0.155)	(0.140)	(0.132)
Age 40 to 49	0.474***	0.183	0.366**	0.925***	− 0.020	0.177
	(0.148)	(0.149)	(0.145)	(0.166)	(0.151)	(0.145)
Age 50 to 59	0.424***	0.240	0.353**	1.155***	− 0.047	− 0.014
	(0.160)	(0.154)	(0.155)	(0.175)	(0.159)	(0.155)
Age 60 and above	0.660***	− 0.002	0.441**	1.234***	0.567**	− 0.050
	(0.227)	(0.212)	(0.216)	(0.253)	(0.250)	(0.208)
Low income	0.392***	0.229*	− 0.075	0.965***	0.401**	0.397***
	(0.133)	(0.133)	(0.144)	(0.183)	(0.168)	(0.134)
Middle income	0.531***	0.195	− 0.165	1.511***	0.663***	0.369**
	(0.146)	(0.144)	(0.154)	(0.191)	(0.176)	(0.145)
High income	0.791***	0.135	− 0.000	2.034***	0.789***	0.529***
	(0.169)	(0.161)	(0.171)	(0.211)	(0.193)	(0.164)
Vocational	− 0.274	0.294*	− 0.066	0.131	− 0.099	− 0.258
	(0.168)	(0.166)	(0.156)	(0.166)	(0.157)	(0.166)
Secondary	− 0.549***	− 0.164	− 0.078	− 0.345***	− 0.023	− 0.344***
	(0.108)	(0.107)	(0.102)	(0.109)	(0.099)	(0.109)
Less than secondary	− 0.349*	0.082	− 0.324*	− 0.546**	− 0.453**	− 0.146
	(0.186)	(0.183)	(0.185)	(0.216)	(0.189)	(0.194)
Single	− 0.167	− 0.121	− 0.113	0.213	0.260**	− 0.255**
	(0.122)	(0.123)	(0.119)	(0.137)	(0.123)	(0.117)
Divorced or widowed	− 0.301	− 0.103	0.094	− 0.280	0.320	− 0.166
	(0.310)	(0.247)	(0.270)	(0.292)	(0.252)	(0.293)
Immediate family	− 0.000	0.086	0.200**	0.011	− 0.090	0.103
	(0.078)	(0.077)	(0.078)	(0.085)	(0.080)	(0.077)
Extended family	− 0.247	0.170	0.408***	0.214	− 0.057	− 0.010
	(0.163)	(0.149)	(0.155)	(0.146)	(0.147)	(0.160)
Chinese	− 0.036	− 0.213***	− 0.149*	0.319***	0.208**	− 0.276***
	(0.089)	(0.082)	(0.082)	(0.089)	(0.086)	(0.083)
Indian	0.092	0.133	− 0.049	0.188	0.075	0.083
	(0.133)	(0.128)	(0.128)	(0.145)	(0.130)	(0.129)
Other ethnic	− 0.425***	0.113	− 0.103	− 0.258	− 0.913***	− 0.054
	(0.146)	(0.157)	(0.151)	(0.193)	(0.178)	(0.139)
Urban	− 0.083	0.174**	0.055	− 0.061	− 0.067	0.067
	(0.091)	(0.089)	(0.087)	(0.092)	(0.086)	(0.089)
Rural	− 0.039	0.250**	− 0.024	− 0.037	0.053	0.148
	(0.105)	(0.102)	(0.102)	(0.111)	(0.099)	(0.106)
North Peninsula Malaysia	− 0.018	− 0.370***	− 0.141	− 0.988***	0.000	0.040
	(0.111)	(0.099)	(0.101)	(0.110)	(0.099)	(0.108)
South Peninsula Malaysia	− 0.034	− 0.781***	− 0.348***	− 0.679***	− 0.141	0.097
	(0.109)	(0.110)	(0.106)	(0.115)	(0.109)	(0.112)
East Peninsula Malaysia	0.602***	− 0.117	0.065	− 0.617***	0.632***	0.406***
	(0.137)	(0.133)	(0.133)	(0.130)	(0.125)	(0.138)
East Malaysia	0.819***	− 0.617***	− 0.441***	− 0.633***	0.760***	0.429***
	(0.132)	(0.127)	(0.129)	(0.158)	(0.146)	(0.128)
/cut1	− 0.664***	− 1.630***	− 1.653***	0.837***	− 1.454***	− 0.728***
	(0.253)	(0.249)	(0.249)	(0.286)	(0.268)	(0.249)
/cut2	2.000***	0.493**	0.163	4.588***	1.330***	0.495**
	(0.252)	(0.248)	(0.248)	(0.295)	(0.265)	(0.248)
Observations	3300	3300	3300	3300	3300	3300

Robust standard errors in parentheses

***p < 0.01; **p < 0.05; *p < 0.1

For financial inclusion, the coefficients of having single bank account and no bank account are negative and significant. This suggest that, in comparison with having several bank accounts, having single bank account and no bank account reduces the probability of being financially resilient and increases the probability of being financially vulnerable. Similar observations are found for resilience components related to keeping control of money, having financial cushion and having financial planning. As for product holdings, a positive and significant relationship is observed between high product holdings and total resilience. This implies that, compared to low product holding, having high product holdings increases the probability of being financially resilient and reduces the probability of being financially vulnerable. Similar findings apply to for the resilience components related to keeping control of money and having financial cushion.

Compared to being female, being male is negatively and significantly related to total financial resilience. This shows that being male lowers the probability of being financially resilient and increases the probability of being financially vulnerable. Similar findings are also observed for male with regard to resilience components related to keeping control of money and handling financial stress. Meanwhile, positive and significant relationship are observed between total resilience and higher age categories. This indicates that being older raises the probability of being financially resilient and lowers the probability of being financially vulnerable. In addition, it also raises the probability of taking a better care of expenditure and having financial cushion. There is a positive and significant relationship between total resilience and higher income categories, demonstrating that higher earning raises the probability of being financially resilient and lowers the probability of being financially vulnerable. Moreover, in results for resilient components, higher earning also raises the probability of having a financial cushion, being able to handle financial shortfall or stress and having a financial planning.

In terms of the relationship between education and total resilience, it is found that being less educated lowers the probability of being financially resilient and raises the probability of being financially vulnerable. Marital status has no bearings on total resilience. As for marital status, compared to married individuals, those who are single are linked to higher probability of handling financial stress but lower probability of having a financial planning. Even though no significant relationship observed between total resilience and supporting family members, supporting immediate and extended family members are associated with greater probability of being able to take care of expenditure. With regard to ethnicity, it is noticed that only the coefficient of other ethnicity is negative and significant. This confirms that, in comparison with being from the Malay ethnic origin, being from the other ethnic origin (other than Indian and Chinese) reduces the probability of being financially resilient and increases the probability of being financially vulnerable. In comparisons to being a Malay, being a Chinese reduces the probability of being able to keep control of money, take care of expenditure, and have a financial planning. On the other hand, it raises the probability of being able to have financial cushion and handle financial shortfall and stress.

Additionally, the findings confirm that place of residence based on the degree of urbanisation do not have any bearing on the probability of being financially resilient. Nevertheless, the findings confirm that, in comparison with living in the city centre, living in the urban or rural areas are linked are associated with greater probability of being able to keep control of money. When it comes to regional location, the findings confirm that in comparison with living in the centre of Peninsula Malaysia, living in the east of Peninsula Malaysia and East Malaysia are associated with higher probability of being financially resilient. Nevertheless, living in the north and south of Peninsula Malaysia, and East Malaysia reduces the probability of being able to keep control of money. Meanwhile, living in the south of Peninsula Malaysia and East Malaysia reduces the probability of being able to take care of the expenditure. It is also observed that in comparison with living in the centre of peninsula Malaysia, living in the rest of the regions lower the probability of having financial cushion. Additionally, the findings confirm that living in the East of Peninsula Malaysia and East Malaysia raise the probability of being able to handle financial stress and having a financial planning.

Additional analysis was done using the five categories of savings as the alternative measure of financial resilience. The findings of the ordered logit regression reported in Table 5 confirm the importance of financial knowledge and financial inclusion in improving savings. The results show that higher financial knowledge and more financial product holdings are associated with the probability of having higher savings. Additionally, being 30 and above and earning higher income are linked to the probability of having higher savings. It is also observed that those with secondary and lower than secondary education are linked with the probability of having lower savings compared to those with university degree. Compared to the Malays, individuals from other ethnic group are associated to the probability of having lower savings. Individuals living in the north, south, and east of Peninsula Malaysia are associated with the probability of having lower savings compared to those living in the centre of Peninsula Malaysia. However, those living is East Malaysia are linked with probability of having higher savings.

**Table 5:** Determinants of the ratio of savings as an alternative financial resilience measure

Variables	(1)
	Savings
	Coeff
Fin knowledge moderate	0.220**
	(0.111)
Fin knowledge high	0.505***
	(0.116)
Single bank account	− 0.083
	(0.085)
No bank account	− 0.120
	(0.228)
Moderate product holdings	0.281***
	(0.095)
High product holdings	0.379***
	(0.112)
Gender	− 0.009
	(0.077)
Age 25 to 29	0.200
	(0.153)
Age 30 to 39	0.617***
	(0.151)
Age 40 to 49	0.570***
	(0.170)
Age 50 to 59	0.584***
	(0.171)
Age 60 and above	0.700**
	(0.274)
Low income	0.692***
	(0.200)
Middle income	1.064***
	(0.209)
High income	1.459***
	(0.221)
Vocational	0.257
	(0.161)
Secondary	− 0.231**
	(0.105)
Less than secondary	− 0.430*
	(0.244)
Single	0.217
	(0.132)
Divorced or widowed	0.269
	(0.407)
Immediate family	− 0.056
	(0.087)
Extended family	0.204
	(0.166)
Chinese	0.111
	(0.092)
Indian	− 0.096
	(0.140)
Other ethnic	− 0.711***
	(0.188)
Urban	− 0.028
	(0.098)
Rural	0.157
	(0.115)
North Peninsula Malaysia	− 0.427***
	(0.121)
South Peninsula Malaysia	− 0.383***
	(0.118)
East Peninsula Malaysia	− 0.472***
	(0.158)
East Malaysia	0.766***
	(0.128)
/cut1	0.459
	(0.306)
/cut2	2.515***
	(0.311)
/cut3	4.004***
	(0.318)
/cut4	5.241***
	(0.342)
/cut5	6.536***
	(0.387)
Observations	2352

Robust standard errors in parentheses

***p < 0.01; **p < 0.05; *p < 0.1

## Discussion and conclusion

Financial resilience of consumers is an essential element for the well-functioning of an economy. Consumers who are financially resilient are able face adverse shock and recover from it by returning to their normal state or having a positive outcome. As such, investigating the factors that contributes to greater financial resilience will not only add value to the existing literature but will also benefit the consumers and policy makers. Malaysia is an emerging country with a growing economy, rising cost of living and high consumer loan. Consumers can be very vulnerable to negative economic shock under this condition. Therefore, it is crucial to understand the determinants of financial resilience among Malaysians. The 2018 OECD (INFE) Financial Literacy nationwide survey is used for this purpose. In particular, the respondents’ total financial resilience and five components of financial resilience as proposed by OECD (2020a; b) are examined. This includes elements related to keeping control of money, taking care of expenditure, having financial cushion, handling financial shortfall and stress, and having a financial planning.

Three main determinants of financial resilience considered are financial knowledge, financial inclusion, and socio-demographic characteristics. Overall, it is found that higher final knowledge increases the probability of being financially resilient. This finding correspond with Kass-Hanna et al. (2021) and Lusardi et al. (2011, 2021), as the results confirm the importance of financial knowledge in improving financial resilience. Furthermore, higher financial knowledge improves all the five components of financial resilience considered in this study. This suggests that higher financial knowledge facilitates individuals in managing their finances and handling financial shortfall and stress.

When comparisons are made with regard to having several bank accounts, it is found that having no bank account or only having a single bank account increases the probability of being financially vulnerable. It is observed that compared to low financial product holdings, high financial product holdings raise the probability of being financially resilient. However, moderate financial product holding financial resilience does not have a bearing on financial resilience when compared to low financial product holdings. The positive impact of financial inclusion measures on financial resilience has been observed in the literature before. Belayeth Hussain et al. (2019) find that individuals in Bangladesh with savings and borrowings accounts are more financially resilient. Lyons and Kass-Hanna (2021) identifies that individual having an individual or a joint account in a formal financial institution in MENA region are better able to come up with emergency funds. Cihak et al. (2016) observe that greater financial product holdings can contribute to financial stability.

Additionally, the results indicate that various socio-demographic characteristics also have impact on financial resilience. It is discovered that men are less resilient compared to women. This is in contrast to studies by Salignac et al. (2021), Belayeth Hussain et al. (2019) and Lyons and Kass-Hanna (2021) observe that women have lower financial resilience. Nevertheless, Daud et al. (2019) find that there is no significant difference in the financial vulnerability of men and women in Malaysia. Older have more financial resilience compared the younger ones. Lusardi et al. (2011) also observe lower financial resilience among younger generations. In line with Belayeth Hussain et al. (2019), Daud et al. (2019), Lusardi et al. (2011) and Salignac et al. (2021), this study finds that those who earn more and have higher education are more resilient. Goyal et al. (2021) argue that individuals with higher income and education have more financial resources and greater awareness about the need to be financially prepared in facing adverse shock, making them more resilient.

Ethnicity’s influence on financial resilience is mixed. More specifically, it is found that those who belong to other ethnic group than Chinese, and Indians are less financially resilient compared to Malays. Variations are also observed among the races in the components of financial resilience. Similarly, Abdullah Yusof (2019) also observed that there is an ethic difference in terms of financial fragility among Malaysians. Differences in financial resilience among the ethnic group is also identified by Lusardi et al. (2011) in the USA. Additionally, compared to individuals living in the central region of Peninsula Malaysia, it is found that those living in the eastern region and East Malaysia have higher financial resilience. Variations are also observed in the components of financial resilience based on the geographical locations. Regional differences in financial resilience were also confirmed by Salignac et al. (2021) in Indonesia with those living in Bali reporting higher resilience compared those living in Central Java, while those living in the rest of regions reporting lower resilience.

## Implications of the findings

This study contributes to the literature by studying the determinants of financial resilience from an emerging economy’s perspective. Individuals’ ability in withstanding an adverse shock by having a good financial management makes them less reliable on state funding and contributes towards greater stability of the financial system. As such, policy makers need to take measures to improve household’s financial resilience. Based on the findings of this study and conclusion derived from the existing literature, the measures taken must consist of elements that integrate financial education with financial inclusion. Development in the financial sector in Malaysia has increased the types of financial products and services with complex structures and terms. Even though the consumers have gained from this through greater product choices, they need to be equipped with sufficient financial knowledge to ensure that they reap the full benefits of greater financial inclusion and are protected from its negative consequences such as over indebtedness.

In line with this, the Financial Education Network (FEN), an inter-agency group co-chaired by Bank Negara Malaysia (BNM) and the Securities Commission (SC), has been established in 2016 with the aim of improving financial literacy among Malaysians via the 5-year National Strategy for Financial Literacy 2019–2023. Among the strategies that have been proposed are to incorporate financial education into the school syllabus and make sure that financial education information is easily accessible and understood. Proper implementation of these strategies is paramount in ensuring that consumers have proactive financial behaviour that can help them persevere during challenging times. Apart from this, the findings here show that there is still room to improve financial inclusion among Malaysians. Financial institutions should take advantage of this through greater marketing of their products and services. However, this needs to be accompanied by appropriate information disclosure that can facilitate consumers decision making. Finally, the findings show financial resilience among Malaysian vary according to their socio-demographic characteristics. As such, policy makers need to ensure that measures taken are designed in a way that all segment of the population, especially the most vulnerable groups, reap the benefits from it.

## Data Availability

We would like to thank Bank Negara Malaysia for providing the access to the Ficancial Capability and Inclusion (FCI) Demand Side Survey Data of 2018. The authors have been given access to Malaysian Financial Capability and Inclusion Demand Side Survey Data by the Central Bank of Malaysia (Bank Negara Malaysia).

## Footnotes

1 The 2018 OECD survey conducted in Malaysia does not include question related to fraud awareness.

2 We acknowledge that this may not necessarily be the case.

3 For example, in the case where Resil 1 = 5.5, Resil 2 = 5, Resil 3 = 3.5, Resil 4 = 6.5, and Resil 5 = 6, the weighted value of Total Resilience = (5.5/11)*53*0.2 + (5/10)*53*0.2 + (3.5/7)*53*0.2 + (6.5/13)*53*0.2 + (6/12)*53*0.2 = 26.5.
